# Dysregulation of the serum and IgG N-glycome in decompensated cirrhosis and its association with Model for End-Stage Liver Disease-Sodium (MELD-Na)

**DOI:** 10.1371/journal.pone.0357709

**Published:** 2026-09-15

**Authors:** Andreea Cislaru, Emre Yagmur, Catherine Bannon, Sinead Cremen, Tom K. Gallagher, Danilo Sarti, Mark W. Robinson, Róisín O’Flaherty

**Affiliations:** 1 Department of Chemistry, Maynooth University, Maynooth, Co. Kildare, Ireland; 2 Kathleen Lonsdale Institute for Human Health Research, Maynooth University, Maynooth, Co. Kildare, Ireland; 3 Department of Biology, Maynooth University, Maynooth, Co. Kildare, Ireland; 4 School of Medicine, University College Dublin, Belfield, Dublin, Ireland; 5 Department of Hepatopancreaticobiliary and Transplant Surgery, St. Vincent’s University Hospital, Elm Park, Dublin, Ireland; 6 Royal College of Surgeons in Ireland, University of Medicine and Health Sciences, Saint Stephen’s Green, Dublin, Ireland; 7 CÚRAM, Science Foundation Ireland Research Centre for Medical Devices, Biomedical Sciences, University of Galway, Co. Galway, Ireland; Pacific Northwest National Laboratory, UNITED STATES OF AMERICA

## Abstract

**Background and Aims:**

N-glycans modulate glycoprotein structure and function and are altered during chronic inflammation. We sought to define the extent of serum and IgG N-glycan disruption in patients with decompensated liver cirrhosis from alcohol-related liver disease (ALD), primary sclerosing cholangitis (PSC), and ALD-related hepatocellular carcinoma (HCC). Finally, we aimed to examine whether serum and IgG glycosylation is associated with changes in Model for End-stage Liver Disease-Sodium (MELD-Na) scores, a clinical marker used to prioritise liver transplantation.

**Methods:**

Serum samples were obtained from patients with ALD (n = 17), PSC (n = 7), ALD-related HCC (n = 4), and healthy controls (n = 10). N-glycans were released, fluorescently labelled, and profiled by hydrophilic interaction ultra performance liquid chromatography (HILIC-UPLC). Chromatograms were integrated into 46 and 23 glycan peaks for serum and IgG respectively. These peaks and their associated glycosylation traits were statistically compared with healthy controls using age- and sex-adjusted linear regression models.

**Results:**

In serum, decompensated cirrhosis shows statistically significant shifts toward less complex, agalactosylated and asialylated biantennary glycans, accompanied by significant losses of highly branched, galactosylated and sialylated structures. IgG mirrored this pattern, which is characteristic of a pro-inflammatory signature, with increased agalactosylation and bisected glycan levels, along with reduced levels of digalactosylated and sialylated species. N-glycan profiles showed significant associations with MELD-Na scores, indicating that inflammatory processes in decompensated liver cirrhosis continue to reshape serum glycoproteins.

**Conclusion:**

Decompensated liver cirrhosis shows profound remodelling of serum and IgG N-glycans. These data establish a reference framework for terminal glycomic disruption in liver disease and highlight the potential value of incorporating glycosylation analysis into broader assessments of liver disease progression.

## Introduction

N-glycosylation is a fundamental post-translational modification, involving the enzymatic attachment of carbohydrate chains to peptides, specifically to Asn-X-Ser/Thr sequences [1]. This highly important process affects protein folding, stability, trafficking, immune recognition, communication and receptor binding [2]. Aberrant glycosylation has been linked to pathophysiology, disease progression and prognostic biomarkers in various conditions including autoimmune disorders, cancers, and rare diseases [3].

Subtle alterations in glycan structures are strongly associated with disease severity and serve as powerful biomarkers [4–7]. For example, altered immunoglobulin G (IgG) galactosylation and sialylation, together with changes in core fucosylation and increased bisecting N-acetylglucosamine (GlcNAc) levels contribute to diagnostic signatures in systemic lupus erythematosus [8,9]. More recently, large-scale population studies have shown that specific serum N-glycan peaks can predict all-cause and cause-specific mortality independently of classical risk factors [10]. Alterations in serum N-glycans are also associated with high-risk drinkers prior to overt liver injury [11]. Studies have found that serum and IgG N-glycosylation have unique signatures in liver disease. Callewaert *et al*. demonstrated that total serum N-glycan profiling can robustly discriminate cirrhotic from non-cirrhotic individuals with high sensitivity and specificity [12]. Subsequent studies have shown that serum N-glycan profiles change progressively with fibrosis stage in viral hepatitis-associated liver disease and can discriminate early and significant fibrosis (F1-F2) from non-fibrotic disease with high diagnostic accuracy prior to the onset of cirrhosis [13]. Many prior IgG glycosylation studies in liver disease concentrate on a single aetiology (commonly viral or autoimmune) and/or examine IgG in isolation [14–16]. Klein *et al*. used immunoglobulin depletion of serum to show that many cirrhosis-associated N-glycan changes co-segregate with Ig fractions, while others reflect liver-derived glycoproteins in fibrosis/cirrhosis cohorts [17]. Taken together these studies clearly demonstrate that glycosylation is a key feature of liver diseases. Its magnitude, specificity and mechanistic significance remain to be understood.

Liver cirrhosis can arise from diverse causes, such as metabolic causes (alcohol-related liver disease (ALD), and metabolic dysfunction-associated steatotic liver disease (MASLD)), genetic causes (lysosomal acid lipase deficiency, and cystic fibrosis), and autoimmune diseases (primary biliary cirrhosis (PBC), autoimmune hepatitis and primary sclerosing cholangitis (PSC)) [18]. Chronic liver injury triggers progressive fibrosis that disrupts the fundamental hepatic architecture, impairing both vascular drainage and metabolic function. As fibrosis advances, normal lobular structures are replaced by regenerative nodules. If untreated, fibrosis progresses to widespread cirrhosis and liver failure, requiring liver transplantation.

The Model for End-Stage Liver Disease-Sodium (MELD-Na) score provides a validated estimate of short-term mortality risk and serves as the main system for prioritising patients for liver transplantation based on medical urgency [19]. MELD-Na is a combination of serum bilirubin, creatinine, international normalised ratio or INR (time required for blood to clot with comparison to normal standards), and sodium levels. Despite its central role in transplantation, MELD-Na has limitations including its tendency to underestimate mortality risk in several clinically vulnerable cohorts and its inability to capture the broader biological complexity of advanced cirrhosis, highlighting the need for a more precise scoring system [20].

Here we analysed the total serum and IgG N-glycosylation in an Irish cohort of 28 liver cirrhosis patients: n = 17 diagnosed with alcohol related liver diseases (ALD), n = 7 diagnosed with primary sclerosing cholangitis (PSC), and n = 4 have hepatocellular carcinoma (HCC) related to ALD. These were compared to healthy donor serum samples, n = 10, where their serum IgG was also isolated as described previously [21,22]. We aimed to characterise the maximum extent of serum and IgG N-glycan alteration in decompensated cirrhosis across this mixed-aetiology cohort, providing a reference profile of N-glycan changes characteristic of a decompensated liver disease. We sought to quantify the contribution of IgG to overall serum glycosylation and define the degree to which immune-derived glycans shape the cirrhotic glycome. This study represents the first paired analysis of total serum and matched IgG N-glycosylation in decompensated liver cirrhosis, and the first such analysis in an Irish cohort. By analysing both fractions from the same individuals, we directly resolve immune-derived versus liver-derived contributions to the cirrhotic glycome. This provides a biologically informed framework for interpreting serum glycosylation changes in advanced liver disease. Finally, we aimed to examine associations between serum and IgG glycosylation and MELD-Na score, to assess whether N-glycosylation changes can stratify the progression of decompensated liver disease.

## Materials and methods

### Chemicals and equipment

Detailed in Supporting Methods 1.1 in S1 File.

### Subjects and ethics

Study participants were undergoing transplant assessment at the Irish National Liver Transplant Centre at St Vincent's University Hospital. Recruitment for this study ran from 2019 to 2021. Eligible patients over the age of 18 years were invited to take part and all disease aetiologies were included. This study received ethical approval from St Vincent's Hospital Cohort Ethics Board (RS19-046) and the Maynooth University Biomedical & Life Sciences Research Ethics Subcommittee (2408611), as per the 1975 Declaration of Helsinki guidelines. All participants provided written informed consent prior to participation. Blood samples were taken at the time of clinical assessment and serum was isolated from serum blood collection tubes. Clinical parameters including age, sex, BMI, ethnicity and MELD-Na scores were recorded for all participants. Following clotting, blood was centrifuged at 1000 RCF for 10 minutes and collected serum was stored in aliquots at -80°C. Control serum was obtained from 10 healthy donors (STEMCELL Technologies).

### N-glycan release and 2-AB labelling of serum using in-gel block method

N-glycan release and labelling from serum samples was performed using an in-gel block method [23]. Serum samples (5 µL) were reduced and alkylated before being embedded into small polyacrylamide gel blocks. After polymerisation for 15 minutes, gels were sectioned into 1 mm^3^ pieces and transferred to an AcroPrep Nucleic Acid binding plate (Pall Life Sciences, PN 8133). N-glycans were released by adding 50 μL PNGase F solution (1:400 in 20 mM NaHCO_3_), followed by an additional 50 μL after 15 minutes, and 50 μL of a NaHCO_3_ buffer, then incubated at 37°C overnight. Released N-glycans were fluorescently labelled with 2-aminobenzamide (2-AB) by adding 5 μL of the mixture to each sample, agitating for 5 minutes, incubating at 65°C for 30 minutes, agitating again, and incubating for an additional 1.5 hours at 65°C. Labelled samples were stored at -20°C for at least 5 minutes before clean-up. Further details are provided in Supporting Method 1.2 in S1 File.

### Automated IgG affinity purification from human serum and N-glycan release

IgG purification was performed using Protein G PhyTips (20 µL resin) on a Hamilton Microlab STAR platform according to the established protocol described in [22]. To optimise yield and purity, several combinations of capture, binding, and elution cycles were tested. SDS-PAGE analysis (Supporting Fig S1 in S1 File) showed that the 6-6-3 cycle configuration (6 aspiration cycles for protein capture and binding and 3 aspiration cycles for elution) produced the highest-purity IgG, with only minimal traces of non-IgG glycoproteins. Isolated IgG was denatured, alkylated and the N-glycans were enzymatically released with PNGase F. N-glycans were labelled with 6-aminoquinolyl-N-hydroxysuccinimidyl carbamate (AQC) as per literature [22]. Full methodological details are available in Supporting Methods 1.3 in S1 File.

### 1D TGX gel electrophoresis

Affinity-purified glycoproteins were reduced, denatured, and analysed by SDS-PAGE using Bio-Rad TGX gels. Samples and a protein ladder were loaded onto a Mini-PROTEAN Tetra Cell system and separated under standard electrophoresis conditions, followed by Coomassie blue staining and destaining to visualise protein purity (Supporting Fig S1 in S1 File). Full procedural details are provided in Supporting Methods 1.4 in S1 File.

### Ultra Performance Liquid Chromatography (UPLC) for N-glycan visualisation and quantification

UPLC analysis of 2-AB labelled serum N-glycans and AQC labelled IgG N-glycans was performed on a Waters ACQUITY UPLC H-Class system, as described previously [21,22]. See Supporting Method 1.5 in S1 File.

### Statistical analysis

All data processing and statistical analyses were performed using R version 4.5.1 (2025-06-13; “Great Square Root”) on a Windows 64-bit platform. The following R packages were used: readxl, dplyr, tidyr, stringr, readr, purrr, tibble, writexl, ggplot2, and knitr. Normality of each glycan variable was tested separately for the healthy and disease cohorts using the Shapiro–Wilk test, data was log-transformed and normalised. P-values were generated using ANOVA (Type I sums of squares) and q-values were generated across multiple glycan variables using Benjamini-Hochberg (BH) correction for serum and IgG N-glycans (Supporting Table S4 and S5 in S2 File respectively), more details provided in Supporting Methods 1.6 in S1 File.

## Results

In this study, we characterised the total serum and IgG N-glycosylation profiles in patients with decompensated cirrhosis to define disease-associated alterations. The cohort is composed of a subset of ALD, ALD-related HCC and PSC cohort from an Irish population (Supporting Table S1 in S2 File).

**Fig 1:**
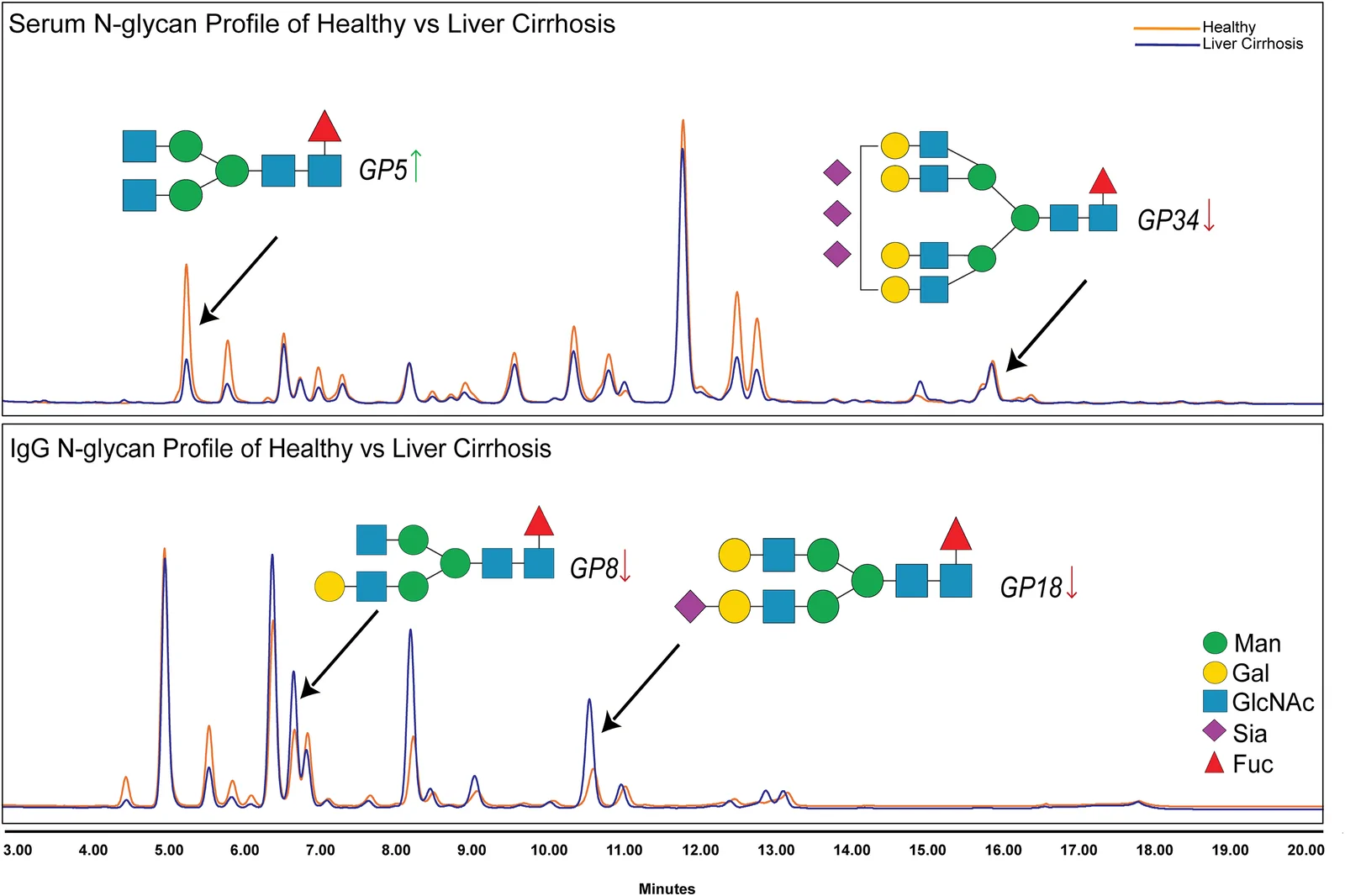
Serum and IgG N-glycan profiles of representative samples for healthy vs liver cirrhosis. The overlaid chromatograms show the serum followed by IgG N-glycan profiles of healthy (orange) and liver cirrhosis (blue). The structures displayed represent the dominant glycan species contributing to that peak and arrows indicate if their levels are increased or decreased in liver cirrhosis. Glycans are depicted with SNFG nomenclature and a legend is provided.

Serum and IgG N-glycans were profiled by hydrophilic interaction ultra-performance liquid chromatography (HILIC-UPLC), yielding 46 serum and 23 IgG glycan peaks (GPs) (Supporting Tables S1 and S2 in S2 File) [22]. The assignment of the major glycan structure(s) underlying each of the 46 serum and 23 IgG GPs is provided in Supporting Table S8 in S2 File. HILIC-UPLC chromatograms demonstrated markedly distinct glycan profiles between healthy controls and patients with cirrhosis (Fig 1). Derived glycan traits, encompassing the relative levels of neutral (S0), sialylation: monosialylated (S1), disialylated (S2), trisialylated (S3), tetrasialylated (S4); galactosylation: agalactosylated (G0, lack of galactose moieties), monogalactosylated (G1), digalactosylated (G2), trigalactosylated (G3), tetragalactosylated (G4); total galactosylation (Gal); oligomannose (also called high mannose); antenna structures: monoantennary (A1), biantennary (A2), triantennary (A3), tetraantennary (A4); fucosylation: core fucose (core F), outer-arm fucose (outer arm F) and sialic acid linkage α2,3-linked (SA (2–3)) and α2,6-linked (SA (2–6)), were calculated and summated according to previously established methods (Supporting Table S3 in S2 File) for serum N-glycans [21], with a subset also calculated following a literature protocol for IgG N-glycans [22].

In healthy populations, both the serum and IgG N-glycome show a systematic variation with age and sex, with consistent shifts in galactosylation, sialylation, branching and fucosylation [6,9,24–26]. Consistent with these findings, linear regression in our cohort (mean age 53 ± 9 yr) showed that older individuals had higher levels of agalactosylated, oligomannose, outer-arm fucosylated and bisected serum GPs, with corresponding decreases in mono-/di-galactosylated biantennary and mono-/tri-sialylated structures (Supporting Fig S2-S3 in S1 File). To further assess the potential influence of age, a sub cohort analysis was performed in participants within the overlapping age range between healthy controls and disease cohorts (healthy: 45-51 years, n = 4; ALD: 45-55 years, n = 7; PSC: 45-55 years, n = 4), as shown in Supporting Fig S9 in S1 File. Directional trends across the majority of serum N-glycan–derived traits were consistent between the full cohort and this age-matched sub cohort, indicating that the observed differences are unlikely to be driven by age distribution and supporting the robustness of the findings. IgG displayed a broadly similar pattern, with increases in agalactosylated and bisected structures and reductions in galactosylated, sialylated and A2-branched GPs. Clear sex associated differences were also observed. Across both serum and IgG (20 males, 8 females overall), females showed a more elaborated N-glycan profile, with higher galactosylation, sialylation and branching, whereas males had more oligomannose and agalactosylated features (Supporting Fig S4-S5 in S1 File). Given these effects, all subsequent disease-related comparisons were adjusted for age and sex.

### Total serum N-glycosylation in the liver cirrhosis cohort

**Fig 2:**
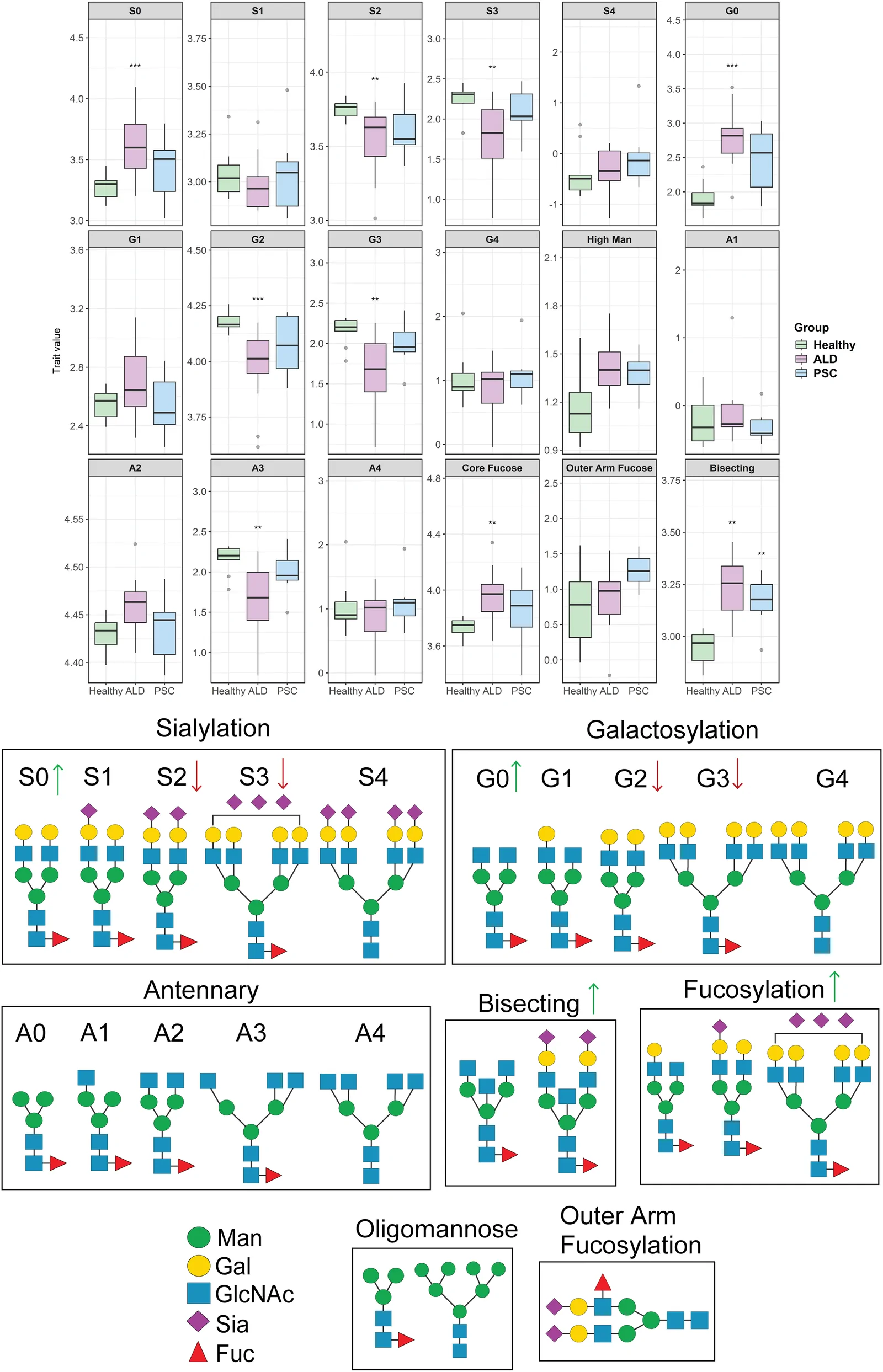
Serum N-glycan traits only. Boxplots showing age- and sex-adjusted serum glycan-derived traits (Supporting Table S3 in S2 File) in healthy (n = 10), ALD (n = 17), and PSC (n = 7). Stars above the boxes indicate the significance of the contrast for each disease cohort versus Healthy (*** p < 0.01, ** p < 0.025, * p < 0.05). Panels on the right show the main glycans associated with each trait that were significantly altered, with arrows indicating whether the levels of corresponding trait is increased or decreased relative to Healthy. The levels of the bisecting glycan trait shows ALD and PSC cohorts are significantly increased compared to Healthy levels. Glycans are depicted with SNFG Nomenclature and a legend is provided.

We examined age and sex adjusted serum GPs and derived glycan traits across the cohort, analysing each disease sub cohort relative to healthy controls, followed by age- and sex-adjusted pairwise comparisons between aetiologies. The full comparison across all 46 serum GPs obtained from HILIC-UPLC glycoprofiles, and derived glycan traits, including healthy, ALD, PSC and HCC subsets is presented in Supporting Fig S6 in S1 File and Supporting Table S4 in S2 File. Fig 2 shows the derived glycan traits across the healthy, ALD, PSC, and HCC cohorts. Multiple GPs were significantly altered compared with the healthy cohort. These included GP4 (p = 0.0044), GP5 (p = 0.00026), GP6 (p = 0.000001), GP7 (p = 0.0021), GP10 (p = 0.0031), GP14 (p = 0.0080), GP15 (p = 0.0441), GP16 (p = 0.0465), GP20 (p = 0.00029), GP25 (p = 0.0337), GP29 (p = 0.0211), GP33 (p = 0.0259), GP34 (p = 0.0189), GP35 (p = 0.0241), GP40 (p = 0.0204), GP42 (p = 0.0147), and GP46 (p = 0.0012). Among the derived glycan traits, significant alterations were observed for S0 (p = 0.0023), S2 (p = 0.0178), S3 (p = 0.0149), G0 (p = 0.000058), G2 (p = 0.0026), G3 (p = 0.0150), A3 (p = 0.0150), core fucosylation (p = 0.0176), and bisecting GlcNAc (p = 0.0111).

Notably the levels of the less complex, lower-branched glycans, including GP4 (A2B), GP5 (FA2), GP6 (M5), GP7 (A2[6]BG1), GP10 (FA2[6]BG1), and GP14 (FA2G2), were significantly increased in ALD compared to healthy controls (Fig 2). In contrast, the levels of the more complex, highly branched and sialylated glycans, such as GP25 (A2G2S(3,6)2), GP29 (A3G3S(3,6)2), GP33 (A3G3S(3,3,6)3), GP34 (A3G3S(3,3,6)3), GP35 (A3BG3S(3,3,3)3), GP40 (A4F1G3S3), GP42 (A4G4S(3,3,3,6)4), and GP46 (A4F3G4S4), were significantly reduced (Fig 2). This shift toward simpler, less sialylated and less branched N-glycan structures reflects impaired glycan processing associated with decompensated ALD. To assess whether glycosylation changes in decompensated cirrhosis converge across aetiologies, we next examined PSC (Fig 2). Significant changes include GP6 (p = 0.0437), GP10 (p = 0.0488), GP15 (p = 0.0440), GP20 (p = 0.0013) and GP29 (p = 0.0053) and the derived glycan trait bisecting GlcNAc (p = 0.0127). The less complex, biantennary glycans and their main glycan structure, such as GP6 (M5), GP10 (FA2[6]BG1), and GP15 (FA2BG2), GP29 (A3G3S(3,6)2) and the bisecting glycan trait showed significantly increased levels, while only GP20 (A2BG2S(6)1) showed significantly decreased levels in PSC when compared to healthy controls. The trends are consistent with those of the ALD cohort.

Additionally, to the ALD cohort, a separate cohort with n = 4 individuals that progressed to ALD-related HCC, were compared to the healthy cohort (see Supporting Fig S6 in S1 File). In the HILIC-UPLC chromatograms several GPs were found to be significant, including GP6 (p = 0.0027), GP10 (p = 0.0232), GP13 (p = 0.0194), GP16 (p = 0.0203), GP24 (p = 0.0246) and GP29 (p = 0.0328), along with the derived glycan traits G3 (p = 0.0405), A3 (p = 0.0405), and bisecting GlcNAc (p = 0.0131). In the ALD-related HCC, we see that the levels of GP6 (M5), GP10 (FA2[6]BG1), GP13 (A2BG2), GP16 (A2[3]BG1S(3)1) are significantly increased compared to healthy controls, whereas the levels of GP24 (A2G2S(3,6)2) and GP29 (A3G3S(3,6)2) are significantly decreased, reflecting again a loss of branching and failure to extend glycan antennae.

### IgG N-glycosylation in the liver cirrhosis cohort

Immunoglobulin G (IgG) is the most abundant glycoprotein in human serum and a major contributor to the total serum N-glycome. As such, we sought to determine whether the observed alterations represented global shifts across the serum glycome or were primarily driven by changes in IgG, the most abundant glycoprotein in circulation [27]. The complete set of IgG profiles, including all 23 GPs and derived traits for healthy against ALD, PSC and ALD-related HCC is presented in Supporting Fig S7 in S1 File.

**Fig 3:**
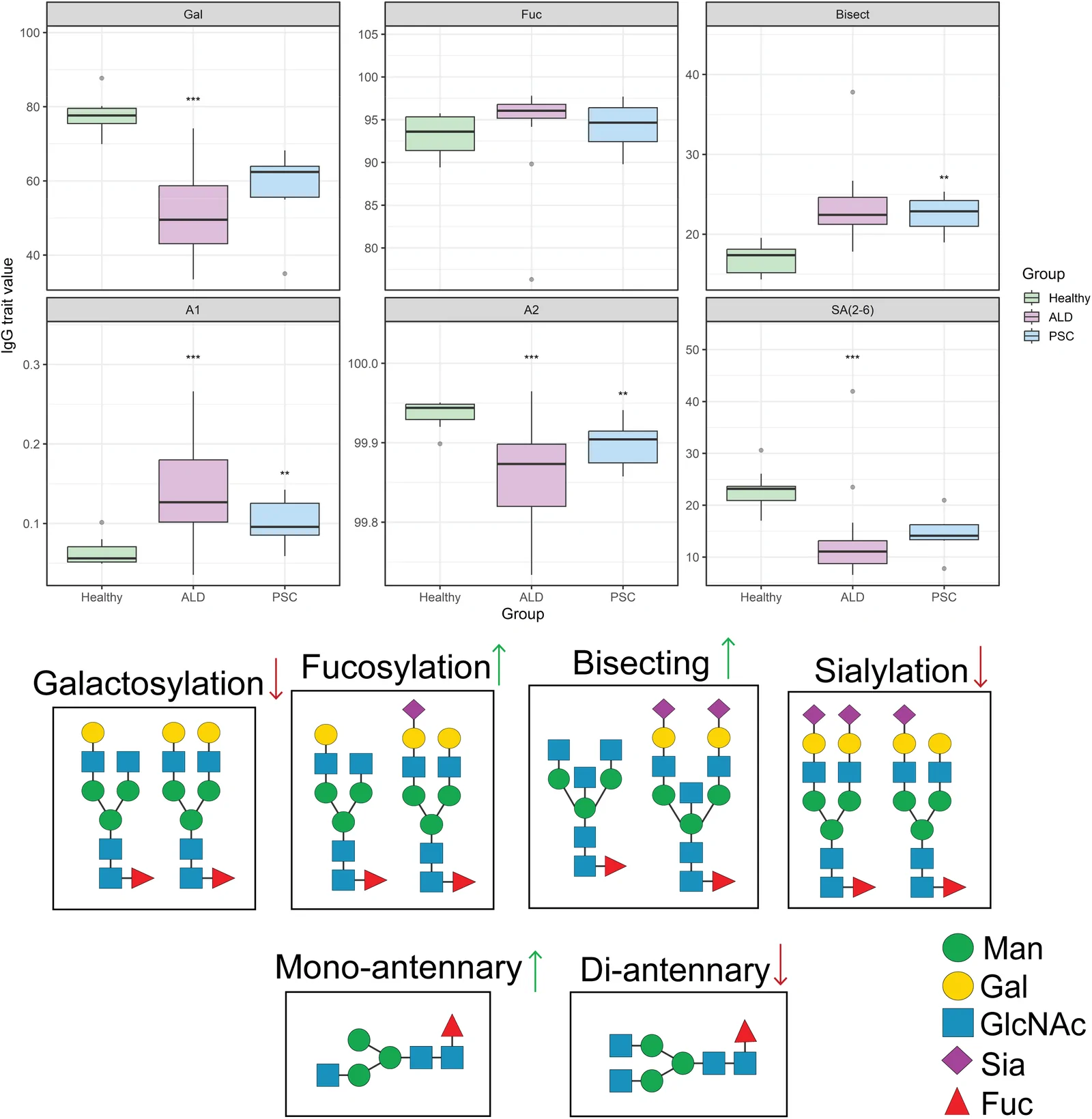
IgG N-glycan traits. Boxplots showing age and sex adjusted serum glycan derived traits (Supporting Table S3 in S2 File) in Healthy (n = 10), ALD (n = 17), and PSC (n = 7). Stars above the boxes indicate the significance of the contrast for each disease cohort versus healthy (*** P < 0.01, ** P < 0.025, * P < 0.05). Only the major glycans associated with each trait are presented which are significantly altered, with arrows indicating whether the corresponding trait is increased or decreased relative to Healthy levels. Glycans are depicted with SNFG Nomenclature and a legend is provided.

Comparing the ALD subset against the healthy cohort, we observed a large number of changes in IgG N-glycans (see Supporting Table S5 in S2 File). These include GP1 (p  = 0.0006), GP3 (p = 0.0001), GP4 (p = 0.00002), GP7 (p = 0.012), GP8 (p = 0.0023), GP10 (p = 0.026), GP11 (0.0428), GP12 (p = 0.0000245), GP13 (p = 0.0228), GP14 (p = 0.015), GP18 (p = 0.000072), GP19 (p = 0.0098), GP20 (p = 0.0045), GP22 (p = 0.0034) and GP23 (p = 0.013). Derived glycan traits were also found to be significantly different, including Gal (p = 0.000042), A1 (p = 0.00067), A2 (p = 0.000671) and SA (2–6) (p = 0.0024) mirroring a similar trend to serum N-glycans (Fig 3).

IgG glycan moieties resemble those found to be significant in total serum N-glycosylation. In the PSC (n = 7) subset, only a small number of IgG GPs reached statistical significance when compared with healthy controls. GP1 (FA1; p = 0.0171), the bisecting GlcNAc trait (p = 0.0140), and the A1 trait (p = 0.0171) were all higher in PSC, whereas the A2 trait (p = 0.0171) was decreased, indicating relatively lower levels of digalactosylated IgG structures (Fig 3). These patterns suggest modest shifts toward more bisected and less galactosylated IgG glycans in PSC consistent with serum N-glycans. Lastly, in the subset of individuals who progressed from ALD to ALD-related HCC (n = 4) (Supporting Fig S7 in S1 File), several IgG glycans were statistically significant when compared with healthy controls. Specifically, GP4 (FA2B; p = 0.00738), GP9 (FA2[6]BG1; p = 0.00769) and the bisecting GlcNAc trait (p = 0.0284) showed higher values, while total IgG galactosylation (Gal; p = 0.00306) was lower (Supporting Table S5 in S2 File).

### Serum and IgG N-glycan alterations across cirrhosis and aetiologies

**Fig 4:**
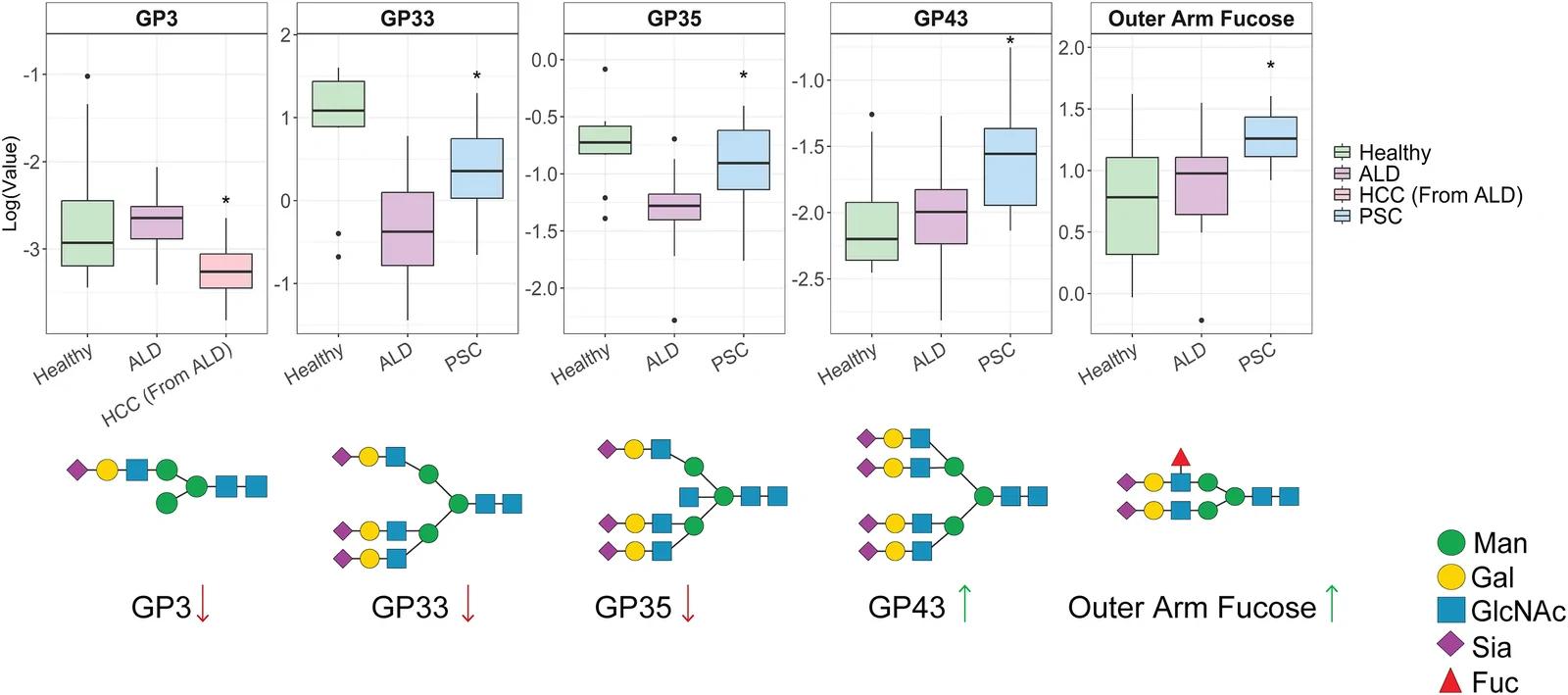
Serum N-glycan peaks significantly altered between ALD (n = 17), PSC (n = 7), and ALD-related HCC (n = 4). Boxplots show age and sex adjusted serum GPs identified as significant in Supporting Table S4 in S2 File. The major glycan structures associated with each glycan peak are depicted. GP33 (A3G3S3), GP35 (A3BG3S3), and GP43 (A4G4S4) levels are increased in PSC compared to ALD, and GP3 (A1[6]G1) levels are decreased in HCC compared to ALD. Glycans are depicted with SNFG Nomenclature and a legend is provided.

To determine whether glycomic alterations in decompensated liver cirrhosis are convergent or aetiology-specific, serum N-glycan peak distributions were compared across clinical sub cohorts ALD versus PSC and ALD versus ALD-related HCC. These comparisons were performed using two-level regression models and the comparator's disease cohort; healthy controls were included only as visual references in Fig 4. Among these comparisons (Fig 4), only GP3 (p = 0.0357) differed significantly between ALD and ALD-related HCC and this peak did not differ between healthy controls and HCC. In contrast, several serum GPs distinguished ALD from PSC, including GP33 (p = 0.0319), GP35 (p = 0.0442), and GP43 (p = 0.0498), along with the outer-arm fucosylation trait (p = 0.0445) (Supporting Table S4 in S2 File). Notably, outer arm fucosylation was not significantly altered between PSC and healthy controls, suggesting that this feature may help differentiate PSC from ALD in advanced cirrhosis; however, the limited PSC sample size requires validation in a larger cohort.

**Fig 5:**
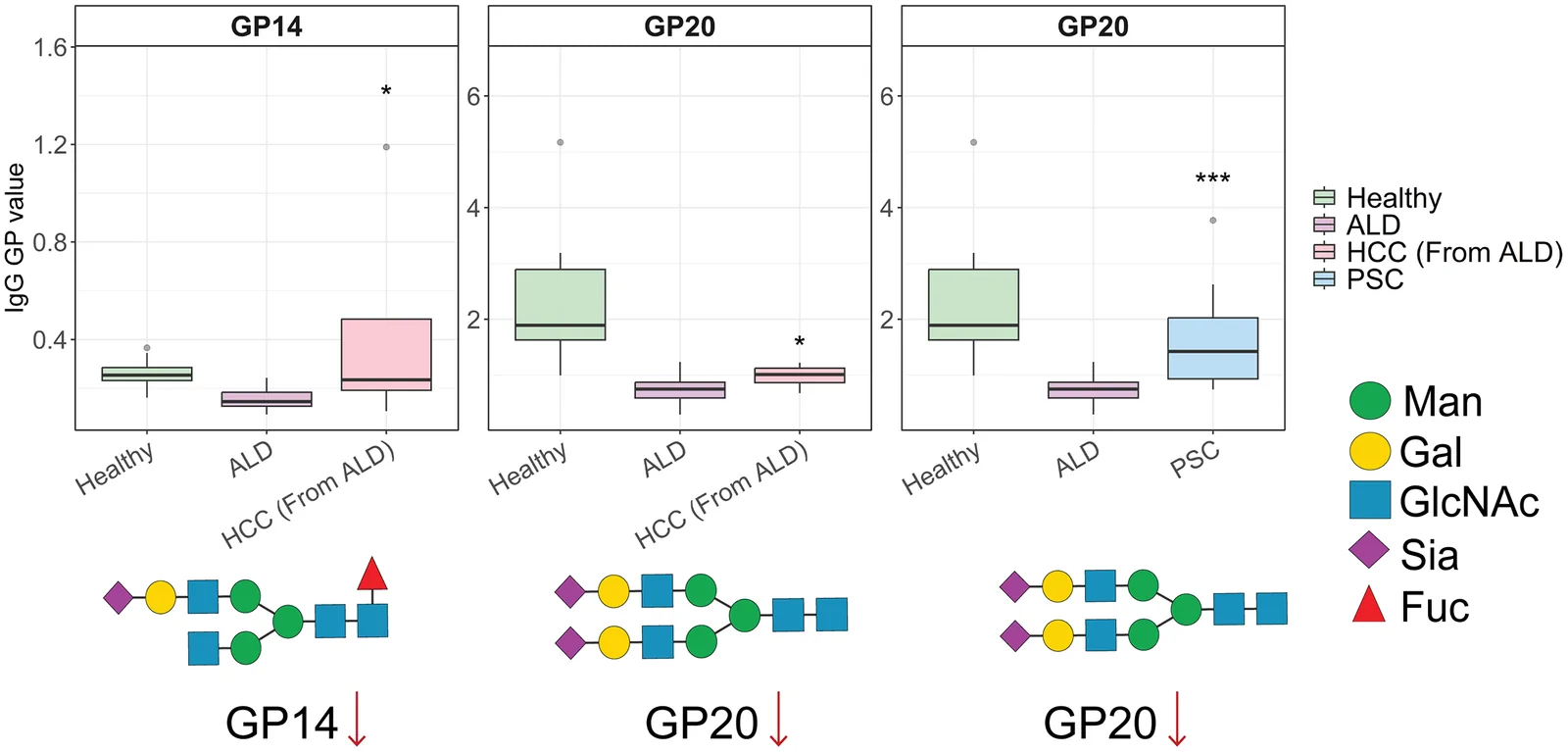
IgG N-glycan peaks significantly altered between ALD (n = 17), PSC (n = 7), and ALD-related HCC (n = 4). Boxplots show age- and sex-adjusted IgG GPs identified as significant in Supporting Table S5 in S2 File. The major glycan structure associated with each glycan peak is depicted. GP14 (FA2[3]G1S(6)1) and GP20 (A2G2S(6,6)2) in HCC have increased levels compared to ALD, and GP20 (A2G2S(6,6)2) has increased levels in PSC compared to ALD. Glycans are depicted with SNFG Nomenclature and a legend is provided.

To complement these findings, we applied the same rationale to IgG N-glycans. We compared IgG GPs across ALD, PSC, and ALD-related HCC (Fig 5). Only GP20 (A2G2S(6,6)2; p = 0.004045) differed significantly between ALD and PSC. In direct comparisons of ALD and ALD-related HCC, two IgG GPs GP14 (FA2[3]G1S(6)1; p = 0.041) and GP20 (A2G2S(6,6)2; p = 0.045) reached statistical significance.

### MELD-Na associated shifts in serum and IgG N-glycosylation

**Fig 6:**
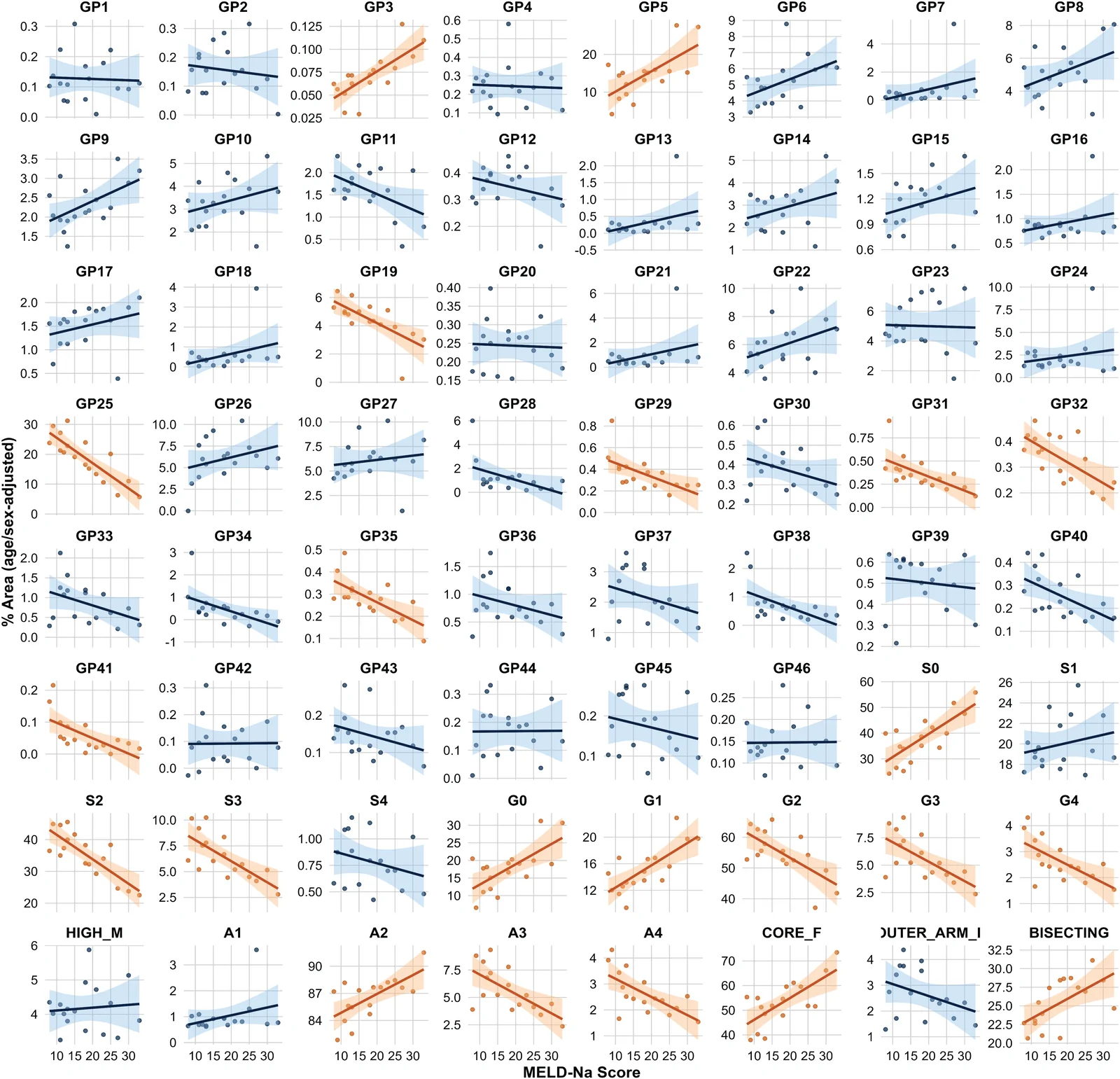
Linear regressions of each individual serum glycan peak (GP1-GP46) and the derived glycan traits of ALD (n = 17) in relation to the MELD-Na score. The highlighted parameters in orange fall within the 20th percentile of glycans and traits that show the strongest positive or negative correlation with MELD-Na.

To further explore the usefulness of total serum and IgG N-glycosylation changes, for clinical application, we examined the correlation between individual GPs and derived glycan traits with the MELD-Na scores (Supporting Table S1 and S2 in S2 File). This analysis was performed on the ALD cohort (n = 17) and on the PSC cohort (n = 7). For the ALD cohort comparing the serum N-glycosylation (GP1-GP46 and derived glycan traits) against their MELD-Na score, we observed positive correlations for GP3 (A1[6]G1), GP5 (FA2), S0, G0, G1, A2 and bisecting indicating that these less complex, asialylated and agalactosylated structures increase with MELD-Na (Fig 6). Negative correlation is seen in GP19 (A2G2S(6]1), GP25 (A2G2S(3,6)2), GP29 (A3G3S(3,6)2), GP31 (A3G3S(3,3)2), GP32 (A3G3S(3,3,3)3), GP35 (A3BG3S(3,3,3)3), and GP41 (A4G4S(3,3,3,3)4), S2, S3, G2, G3, G4, A3, and A4. This suggests that more complex, highly sialylated, galactosylated, and branched (tri- and tetra-antennary) GPs decrease with increasing MELD-Na score. Full regression statistics for ALD versus MELD-Na for serum and IgG N-glycans are reported in Supporting Table S6 in S2 File.

The linear regression models for IgG N-glycosylation against the MELD-Na score were modest compared to serum for the ALD cohort (Supporting Fig S8 in S1 File). GP3 (FA2), showed a mild increase in levels with rising MELD-Na, while GP7 (FA2[6]G1) and GP8 (FA2[3]G1) decreased slightly. GP21 (A2BG2S(6,6)2) showed a small upward trend, whereas total galactosylation declined and α2,6-linked sialylation increased with worsening liver function. Although the direction of change mirrored those seen in serum, IgG slopes were shallower, indicating that IgG glycans shift only gradually with MELD-Na, in contrast to the stronger, more dynamic serum glycan responses.

To facilitate exploration of glycosylation changes across relative disease severity within the observed cohort, MELD-Na values were stratified into three distribution-based cohorts (low: 8-15, mid: 16-25, and high: 26-33). These cohorts served as an exploratory analytical framework to visualise and compare glycosylation features across lower, intermediate, and higher positions along the MELD-Na continuum. Using this approach in the combined cohort (ALD and ALD-related HCC, n = 21), we evaluated individual serum GPs and derived glycan traits for their ability to discriminate relative positions along the MELD-Na scale. Age- and sex-adjusted one-versus-rest area under the curve (AUC) analyses were first assessed across the three cohorts (Supporting Table S7 in S2 File). Several serum GPs demonstrated strong discriminative performance (AUC ≥ 0.90) for the high cohort [26–33], including GP2 (FA1), GP8 (FA2[6]G1), GP18 (FA2[3]BG1S(3)1), GP24 (A2G2S(3,6)2), GP29 (A3G3S(3,6)2), S1, G1, and G2 representing the most distinctive glycosylation features associated with more severe disease. In contrast, a broader set of serum GPs and traits (GP2 (FA1), GP10 (FA2[6]BG1), GP18 (FA2[3]BG1S(3)1), GP24 (A2G2S(3,6)2), GP25 (A2G2S(3,6)2), GP27 (FA2G2S2(3,6)2), GP32 (A3G3S(3,3,3)3), GP33 (A3G3S(3,3,6)3), GP45 (A4F1G4S(3,3,3,6)4), GP46 (A4F3G4S(3,3,3,3)4), S1, S2, G0, G1, G2, A1, A2, and outer arm fucosylation) showed moderate discriminative ability (AUC 0.70-0.78) for the low cohort (MELD-Na 8-15). For the mid cohort (MELD-Na 16-25), they exhibited limited discriminatory power, with GP14 (FA2G2) the only feature reaching an AUC of approximately 0.70. This stepwise shift in glycosylation across MELD-Na stratification is consistent with progressive alterations in the underlying glycan biosynthetic pathways as liver disease severity increases.

**Fig 7:**
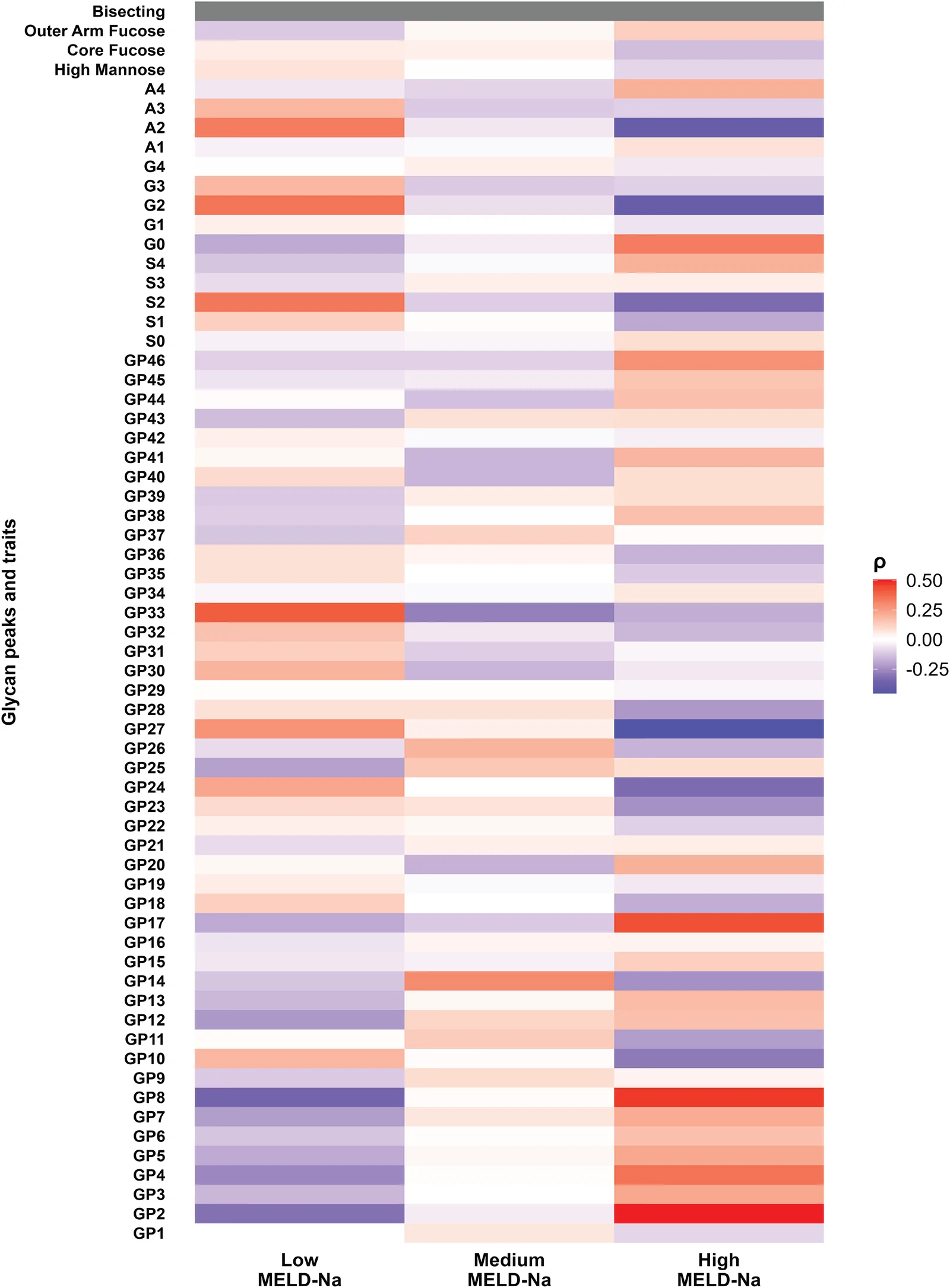
This heatmap displays the Spearman rank correlations between all serum glycan peaks (GP1-GP46), all derived glycan traits, and MELD-Na severity bands. Correlations were calculated using a band-vs-rest approach in which the target MELD band was coded as 1 and all other individuals as 0. All glycan values were log10-transformed prior to analysis to stabilise variance and improve comparability across glycan structures. The colour scale reflects both the strength and direction of association, with red indicating glycans enriched in each MELD-Na band, blue indicating those reduced in that band, and white representing minimal differences. Cells shown in grey indicate NA values.

Distinct glycomic patterns were observed across the MELD-Na spectrum. The MELD-Na strata were assessed using a band-versus-rest Spearman correlation approach (Fig 7). The low MELD-Na cohort [8–15] was characterised by relative depletion of several low-complexity and fucosylated serum GPs, including GP2 (FA1), GP3 (A1G1), GP4 (A2B), GP5 (FA2), GP7 (A2BG1), GP8 (FA2G1), GP16 (A2[3]BG1S(3)1), GP25 (A2G2S2), GP38 (A4G4S(3,3,3)3), GP45 (A4F1G4S3) and GP46 (A4F3G4S4), together with reduced agalactosylation (G0), tetra-antennary structures (A4), and outer-arm fucosylation. In contrast, this cohort showed relative enrichment of more processed glycans, including GP18 (A2G2S1), GP24 (A2G2S2), GP29-GP35 (predominantly tri-antennary, fully galactosylated and highly sialylated structures, e.g., GP33: A3G3S3; GP35: A3BG3S3), GP40 (A4F1G3S3), alongside higher levels of mono-, di-, and trisialylated traits (S1-S3) and mono- to trigalactosylated traits (G1-G3), reflecting more complex type glycans. The medium MELD-Na cohort [16–25] showed generally weaker correlations overall and show less pronounced band-specific changes. Relative depletion was observed for serum GP33 (A3G3S3), GP37 (A3F1G3S(3,3,3)3), S2, S3, G3, and A3, while modest enrichment was seen for GP9 (FA2[3]G1), GP11 (M6 D3), GP14 (FA2G2), GP16 (A2[3]BG1S(3)1), GP25 (A2G2S2), GP26 (A2BG2S(3,6)2), GP45 (A4F1G4S3), as well as the mono- and tetra-antennary traits (A1, A4) and outer-arm fucosylation. In contrast to the low MELD-Na cohort, the high MELD-Na cohort [26–33] exhibited a marked depletion of more complex and extended glycan structures, including GP18 (A2G2S1), GP24 (A2G2S2) and GP28-GP35 (predominantly tri-antennary, fully galactosylated and sialylated species), as well as reduced S1, S2 and G1 traits. Conversely, this cohort was enriched for lower-complexity glycan features, including GP2 (FA1), GP4 (A2B), GP5 (FA2), GP6 (M5), GP7 (A2BG1) and GP8 (FA2G1), alongside increased agalactosylation (G0) and asialylation (S0) levels. This pattern represents the reciprocal glycomic profile to that observed in the low MELD-Na cohort.

## Discussion

This study set out to characterise how serum and IgG N-glycosylation changes in decompensated liver cirrhosis, to assess the contribution of IgG to the overall serum glycome in the context of decompensated liver disease, and to determine whether different aetiologies produce distinct glycomic signatures. Expanding on this, we explore how serum and IgG N-glycosylation correlate with MELD-Na score.

Using HILIC-UPLC analysis we characterised serum N-glycosylation in decompensated liver cirrhosis and observed a shift toward less complex, less branched, and less sialylated structures compared to healthy controls, alongside a reduction in highly processed multi-antennary glycans (Supporting Fig S6 in S1 File). We also see this trend in their IgG N-glycome (Supporting Fig S7 in S1 File). In ALD (n = 17), we observed increases in agalactosylated and bisected biantennary structures and corresponding decreases in galactosylated and sialylated glycans, a pattern reflected by highly significant p- and q-values across multiple IgG GPs (Supporting Table S5 in S2 File). This indicates that IgG contributes substantially to the serum glycosylation signature associated with ALD.

Alcohol consumption and chronic liver disease are closely linked, with sustained alcohol exposure representing a major aetiological driver of hepatic injury and cirrhosis worldwide. However, the glycomic impact of alcohol is likely to be stage-dependent and influenced by current exposure versus advanced hepatic dysfunction [28,29]. In a large general population cohort (n = 1516), we previously showed that risk alcohol intake was associated with a characteristic serum N-glycomic signature, including higher abundances of tri-antennary, tri-galactosylated, and tri-sialylated structures (O'Flaherty et al, Biomoelcules 2022). In contrast, in the current cohorts, we observed lower abundances of these higher order glycans (Fig S4 in S1 File).

At a cellular level, alcohol-associated hepatic injury and cirrhosis provide a mechanistic rationale for persistent glycomic disruption, even in the absence of ongoing ethanol exposure. Glycosyltransferases involved in galactosylation, sialylation, and N-glycan branching including galactosyltransferase, sialyltransferase, and N-acetyllactosaminide β-1,6-N-acetylglucosaminyltransferase (a branching GlcNAc transferase) have been measured in human serum and were 35-55% lower in heavy drinkers compared to healthy controls [30]. Consistent with this, Blomme *et al.* demonstrated alcohol-induced Golgi damage and mislocalisation of N-acetylglucosaminyltransferases in hepatocytes, leading to reduce N-glycan branching and altered N-glycans [31]. Individuals in the present cohort were abstinent at the time of sampling, indicating that the observed reduction in glycan complexity is unlikely to reflect direct effects of ongoing alcohol consumption. While partial recovery of glycosylation capacity has been reported following alcohol cessation in early-stage disease, available evidence suggests that these alterations may persist in advanced cirrhosis [32–34]. Notably, GP5 (FA2), an agalactosylated, fucosylated biantennary glycan that was significantly increased in ALD, has also been established as an age-associated serum N-glycan feature. In our previous population-based analysis of age-related serum N-glycome variation in men and women, agalactosylated and lower-complexity glycan features were associated with ageing and comorbidity [24]. While the present analyses were adjusted for age, the pronounced elevation of GP5 (FA2) in ALD may therefore reflect convergence between disease-associated inflammatory remodelling and age-related glycosylation pathways.

In ALD-related HCC, the serum N-glycosylation additionally shows a shift towards less processed GPs, specifically GP6 (M5), GP10 (FA2[6]BG1), GP13 (A2BG2) and GP16(A2[3]BG1S(3)1). This is accompanied by a corresponding decreased level in the more processed GPs, like GP24 (A2G2S(3,6)2) and GP29 (A3G3S(3,6)2). This part of the study is limited due to the small samples size and requires a larger validation study. RNA-seq-based glycogene expression profiling studies in HCC have demonstrated coordinated dysregulation of the N-glycosylation biosynthetic pathway, including increased expression levels of early-pathway enzymes, e.g., ALG enzymes, mannosidases, and MGAT1. Disruption of later-stage processing enzymes responsible for branching, elongation, and terminal modification include, MGAT2/4/5, galactosyltransferases, fucosyltransferases, and sialyltransferases. This mechanistically supports the accumulation of less processed and incompletely extended glycans observed in our serum data [35]. Furthermore, functional evidence supports the role for dysregulated glycosylation enzymes in HCC progression, MGAT4A, a key N-glycan branching enzyme, is upregulated in HCC tissue, and its knockdown diminishes a tumour-associated N-glycan structural feature and suppresses HCC cell invasion and migration, supporting a functional role for altered glycosylation in aggressive tumour behaviour. [36].

In serum, PSC was compared to healthy controls and showed significant shifts resembling those in ALD, including increases in the levels of several less complex biantennary glycans and higher levels of bisecting GlcNAc, with only one sialylated structure with observed reduced levels. IgG showed fewer significant alterations overall, specifically GP1 (FA1), A1, A2 and bisecting. Together, these findings indicate a shared shift toward more bisected and less galactosylated structures across serum and IgG in both ALD and PSC subsets. Site-specific plasma glycoproteomic studies have identified PSC-associated N-glycan alterations compared with healthy controls, characterised by reduced galactosylation, increased bisecting GlcNAc levels, and a shift toward less complex biantennary structures, consistent with disease associated glycan remodelling [37]. Although direct evidence linking ER stress to Golgi dysfunction and specific glycosylation enzyme mislocalisation in PSC liver tissue is currently lacking, bile acid accumulation has been shown to induce ER stress in hepatobiliary cells, highlighting the need for further investigation of downstream effects on glycosylation pathways [38,39].

In a healthy population, the serum N-glycome is sex dependent, showing that tri-antennary and tri-galactosylated N-glycans are more abundant in women than in men [24]. A similar pattern is observed for IgG, where women exhibit higher levels of IgG galactosylation and sialylation compared with men. [40]. Additionally, women develop alcohol-associated liver disease (ALD) at lower levels of alcohol consumption, whereas men are overrepresented among ALD cases and typically have higher lifetime alcohol exposure. [41]. In our study, in ALD cohort, females were enriched for bisected, lower-antennary, and lower-sialylation traits (A1, A2, S0 and S1), whereas males showed higher relative abundances of highly branched and highly sialylated glycans (A3, A4, S3 and S4), indicating a greater loss of glycan complexity in females. In our PSC cohort, sex differences were less pronounced and primarily involved modest increases in bisecting and galactosylation traits in females (Supporting Fig S4 in S1 File). Importantly, all statistical models were adjusted for sex, indicating that the observed glycomic differences are not solely attributable to sex-related effects. In work done by our cohort, a general population cohort of heavy drinkers showed increased glycan branching, galactosylation and sialylation levels, particularly in women, reflecting an early alcohol-associated glycomic response in the absence of clinically overt liver disease. [11]. These changes likely represent a physiological and potentially reversible adaptation, as they occur before measurable liver dysfunction and may normalise with alcohol cessation. Although γ-glutamyl transferase (GGT) was increased in a dose-dependent manner with alcohol intake in this study, levels largely remained within normal physiological ranges, indicating that glycomic changes precede hepatic injury. In contrast, the present study captures the decompensated stage of ALD, where glycomic remodelling is no longer possible. At this advanced stage females showed a more pronounced shift toward simplified, bisected and less sialylated glycan features, consistent with a greater loss of glycan complexity at advanced disease stages. Taken together, these observations raise the possibility that glycomic deterioration may occur more rapidly in women. Further studies capturing glycomic changes at an early disease stage and incorporating sex-stratification could help bridge this gap.

Given that the serum N-glycome integrates contributions from both inflammatory and liver-derived acute-phase proteins, we specifically examined IgG as a major inflammatory glycoprotein. However, the observed trends in the serum N-glycome cannot be attributed to IgG alone. Several abundant hepatic glycoproteins- including haptoglobin (Hpt), transferrin (Trf), α1-acid glycoprotein (AGP), and complement components C3 and C4 [42–45] -as well as inflammatory glycoproteins such as IgA1 [46], are also likely to contribute to the overall serum N-glycosylation profile. Liver-derived acute-phase proteins, including AGP, A2M, C3, and C4, normally supply multi-sialylated tri- and tetra-antennary (A3/A4) glycans to serum but decline in abundance or become structurally altered in advanced liver failure [47]. These observations are consistent with the reduced levels of A3 and A4 glycan structures observed in our data owing to that fact that serum contains many proteins including those mentioned above that may be decorated with such higher-order glycans, which cannot be derived from IgG, an exclusively biantennary glycoprotein [48]. Additionally, several abundant liver-derived glycoproteins such as zinc-α2-glycoprotein, vitronectin, fetuin-A and ceruloplasmin are implicated in liver cirrhosis yet remain largely under-studied in terms of their glycosylation and warrant further investigation [49–51].

Primary sclerosing cholangitis (PSC) is an immune-driven liver disease, and previous studies have demonstrated particularly pronounced disease-associated glycosylation changes in IgG. Analyses of IgG Fc glycosylation in PSC indicate a subclass-dependent pattern, with IgG1 exhibiting the most marked alterations, characterised primarily by reduced Fc galactosylation, alongside subclass-specific changes in sialylation and, in some studies, increased levels of bisecting GlcNAc, compared with other IgG subclasses [52]. In the present study, which profiles total IgG N-glycosylation rather than subclass-resolved Fc glycans, fewer glycan features reached statistical significance in PSC. Nevertheless, the observed alterations in GP1 (FA1), bisecting GlcNAc, and the A1 and A2 traits indicate a consistent IgG glycosylation signature. This analysis captures the global IgG N-glycome rather than a focus on a subclass specific IgG1.

With the major alterations in the serum and IgG glycoprofiles observed, we next assessed whether distinct aetiologies display unique glycosylation features (ALD, PSC, and ALD-related HCC). In serum, comparison of ALD and ALD-related HCC identified only GP3 (A1[6]G1) as significantly different, indicating that HCC-associated glycomic changes in advanced ALD are limited and largely overlap with those of cirrhosis alone. In contrast, PSC and ALD were distinguished by three significant glycan features, including GP33 (A3G3S3), GP35 (A3BG3S3), GP43 (A4G4S4), and the outer arm fucosylation trait. Outer-arm fucosylation differs between ALD and PSC, while remaining unchanged between PSC and healthy controls, suggests that this alteration may be specific to ALD-related decompensation rather than a shared feature of advanced liver disease (Fig 4). Interestingly, site-specific glycopeptide analyses of liver-secreted proteins including hemopexin and complement factor H have demonstrated that core and outer-arm fucosylation are differentially expressed across individual glycosylation sites and proteins in liver diseases [53]. Outer-arm fucosylation shows disease-associated increases at specific glycosites of hemopexin, whereas core fucosylation is undetectable at certain glycosites of complement factor H regardless of disease severity [42]. This site- and protein-specific microheterogeneity suggests that fucosylation patterns in liver disease are shaped by factors beyond general upregulation of fucosyltransferase activity [54]. Our observation that outer-arm, but not core, fucosylation distinguished ALD from PSC, while core fucosylation was significantly altered in ALD relative to healthy controls but did not differentiate between the two aetiologies, raises the possibility that the aetiology-specific signal reflects differential fucosylation at individual glycosylation sites or specific liver-derived glycoproteins, as described for hemopexin and complement factor H. Such site resolved analyses may offer a more granular understanding of how outer-arm fucosylation differs between ALD and PSC.

IgG glycosylation shows significance in GP20 (A2G2S(6,6)2) a galactosylated glycan peak. While GP14 (FA2[3]G1S(6)1) and GP20 (A2G2S(6,6)2) differentiated ALD-related HCC from ALD (Fig 5). Previous work shows that glycans can differentiate liver disease aetiologies. A tri-fucosylated tetra-antennary glycan on AGP, for example, distinguishes HCC from cirrhosis across ALD, non-alcoholic steatohepatitis (NASH) and hepatitis C virus (HCV) infection, achieving AUCs of ~0.70-0.75 and improving performance when combined with alpha-fetoprotein (AFP) and the international normalized ratio (INR) [55]. Our findings suggest that whole-serum and IgG glycosylation provide complementary discriminatory value beyond these existing biomarkers.

Lastly, our work investigated how serum and IgG N-glycan profiles vary along the MELD-Na spectrum in decompensated ALD. We stratified the full cohort into three MELD-Na ranges to identify the glycan features that define each severity stage-the stratifications included low: 8-15, mid: 16-25, and high: 26-33. We found that numerous serum GPs and glycan traits correlate strongly with MELD-Na. Since many serum glycoproteins depend on the liver for their synthesis and glycan maturation, worsening MELD-Na scores are reflected by a parallel loss of serum glycan complexity as opposed to IgG which showed less pronounced associations with MELD-Na. Despite its weak association with MELD-Na (Supporting Fig S8 in S1 File), IgG distinguished ALD from healthy in a statistically robust manner. The stronger association of serum N-glycosylation with the MELD-Na is expected, as MELD-Na reflects circulating serum measures, whereas IgG glycosylation captures a distinct immune-derived component. Together, these findings suggest that serum and IgG N-glycans capture complementary biological dimensions of ALD: the serum glycome reflects the extent of hepatic functional decline, whereas the IgG glycome reports on the underlying inflammatory environment. Previous work has linked individual glycoprotein glycoforms, such as haptoglobin and M2BPGi, with MELD scores [56]. Sanda et al. showed that specific tri- and tetra-antennary fucosylated haptoglobin glycoforms (e.g., A3G3F1, A4G4F1) correlate with higher MELD scores and lower platelet counts in cirrhosis and HCC [57]. To our knowledge, no prior study has systematically related global serum and IgG N-glycome traits to MELD-Na in an ALD decompensated cohort. Likewise, associations between global serum and IgG N-glycosylation and MELD-Na have not previously been systematically examined across liver disease aetiologies.

Examining N-glycosylation patterns across the MELD-Na scale provides additional insight into how the serum N-glycome progressively deteriorates with worsening hepatic decompensation. Some modifications of the MELD-Na have been proposed in the past. MELD-3.0 was introduced to correct sex-related bias arising from lower creatinine levels in females, reinforcing the need for additional biological markers [58]. In our hands, with the three groups, termed low, mid and high we observed differences in their glycosylation. The low MELD-Na cohort showed increased levels of lower-complexity agalactosylated and asialylated glycans suggesting that hepatic dysfunction is present. The cohort termed mid showed a cluster of upregulated low-complexity peaks, but with modest discriminatory ability, possibly reflecting the changes that take place biosynthetically as liver cirrhosis worsens. In contrast, the high MELD-Na range represented a complete collapse in structural complexity. These findings show that the serum N-glycome transitions from simpler glycan motifs toward a near-complete breakdown of glycan processing as decompensation worsens (Fig 7 and Supporting Table S7 in S2 File). As glycomic deterioration reflects both hepatic synthetic failure and systemic inflammation, these dimensions are not fully captured by MELD-Na alone. Integrating glycan markers could provide clinicians with earlier or more granular insight into the disease trajectory and decisions about liver transplant.

A limitation of this study is the use of commercially sourced healthy controls, which may differ from the patient cohort in terms of ethnicity and lifestyle factors. While we acknowledge this, the glycomic changes observed in the present study, including reductions in galactosylation, sialylation, and glycan branching in decompensated liver disease, are consistent in direction with those reported in the published literature for comparable liver disease populations [59].

## Conclusion

In this study we provide a detailed characterisation of serum and IgG N-glycosylation in Irish decompensated liver cirrhosis, demonstrating that glycomic remodelling accompanies advanced hepatic dysfunction. The serum N-glycome shows a marked shift toward less sialylated, less galactosylated, and less branched structures, while IgG exhibits a pronounced inflammatory signature characterised by increased agalactosylation and bisecting GlcNAc, reflecting the immune-driven component of disease.

These findings can be placed within a broader disease continuum: whereas earlier population-based studies of heavy drinkers without overt liver disease showed increased glycan branching, galactosylation and sialylation consistent with a reversible, compensatory stress response, the present study captures the opposite extreme. In decompensated cirrhosis, particularly at higher MELD-Na scores, glycan complexity collapses, suggesting a transition from adaptive to non-reversible glycomic disruption. A key unanswered question is at what stage this transition occurs.

We further show that serum glycan profiles correlate strongly with MELD-Na and can discriminate between severity ranges, suggesting that glycan features could complement MELD-Na in assessing disease progression. Future work should incorporate sex-stratified designs and larger, more aetiologically diverse cohorts, and extend profiling to additional serum proteins to clarify the upstream drivers of these changes.

## Supporting information

S1 File Supporting methods and figures. (DOCX)

S2 File Supporting tables. (XLSX)

S1 Fig Graphical abstract. (TIF)
