# Supplementary material for: Dysregulation of the serum and IgG N-glycome in decompensated cirrhosis and its association with Model for End-Stage Liver Disease-Sodium (MELD-Na)
Source: PLoS One. 2026 Sep 15;21(9):e0357709. doi: 10.1371/journal.pone.0357709 (PMC13577341; doi:10.1371/journal.pone.0357709)
Supplement: Supplementary file 1 [file pone.0357709.s001.docx]

**Supporting Methods and Figures**

## Dysregulation of the Serum and IgG N-Glycome in Decompensated Cirrhosis and Its Association with Model for End-Stage Liver Disease-Sodium (MELD-Na)

Andreea Cislaru^1^, Ismail E. Yagmur^1,2^, Catherine Bannon^2,3^, Sinead Cremen^4^, Tom K. Gallagher^5^, Danilo Sarti^6^, Mark W. Robinson^2,3^, Róisín O’Flaherty^1,3,7*^

^1^Department of Chemistry, Maynooth University, Maynooth, Co. Kildare, Ireland, W23 F2H6. ^2^Kathleen Lonsdale Institute for Human Health Research, Maynooth University, Maynooth, Co. Kildare, Ireland, W23 F2H6. ^3^Department of Biology, Maynooth University, Maynooth, Co. Kildare, Ireland, W23 F2H6. ^4^School of Medicine, University College Dublin, Stillorgan Road, Belfield, Dublin 4, Ireland. D14 K5X0. ^5^Department of Hepatopancreaticobiliary and Transplant Surgery, St. Vincent’s University Hospital, Elm Park, Dublin 4, Ireland. D04 T6F4. ^6^Royal College of Surgeons in Ireland, University of Medicine and Health Sciences, 123 Saint Stephen’s Green Dublin 2, Ireland. D02YN77. ^7^CÚRAM, Science Foundation Ireland Research Centre for Medical Devices, Biomedical Sciences, University of Galway, Co. Galway, Ireland. H91 W2TY.

*Correspondence: +353 1 708 6477, [roisin.oflahery@mu.ie](mailto:roisin.oflahery@mu.ie)

**Keywords:** *N*-glycosylation; Serum; Liver Cirrhosis; IgG; MELD-Na Score

Table of Contents

[Dysregulation of the Serum and IgG N-Glycome in Decompensated Cirrhosis and Its Association with Model for End-Stage Liver Disease-Sodium (MELD-Na)	1](#_Toc238626498)

[Supporting Methods	2](#_Toc238626499)

[1.1. Chemicals and equipment	2](#_Toc238626500)

[1.2. N-Glycan release and 2-AB labelling of serum using in-gel block method	3](#_Toc238626501)

[1.3. Automated IgG affinity purification from human serum and N-glycan release	4](#_Toc238626502)

[1.4. ^1^D TGX gel electrophoresis	5](#_Toc238626503)

[1.5. Ultra Performance Liquid Chromatography (UPLC) for N-glycan visualisation and quantification	5](#_Toc238626504)

[1.6. Statistical analysis	6](#_Toc238626505)

[Supporting Figures	6](#_Toc238626506)

# Supporting Methods

### 1.1. Chemicals and equipment

Mili-Q water used for all buffer preparations and washing steps. Milli-Q water was generated using a Millipore Milli-Q Integral 3 A10 TOC system. Pre-packed 300 µL tip columns containing 20 µL Protein G resin were obtained from Phynexus. Plates and consumables including 96-well Greiner plates, Nunc plates, robotic flat-bottom reservoirs, Nanosep Omega 10 kDa centrifugal devices, and HyperSep-Diol 100 mg well plates were purchased from ThermoFisher Scientific. UPLC analysis of 2-AB–labelled samples and dextran were performed using a Waters ACQUITY UPLC H-Class system equipped with an ACQUITY UPLC Glycan BEH Amide column from Waters. HPLC micro-vials (0.3 mL PP short-thread) were purchased from Apex Scientific. Acetonitrile (HPLC grade), methanol (HPLC grade), and ethanol were purchased from Honeywell. Reagents including dithiothreitol (DTT), iodoacetamide (IAA), sodium bicarbonate (SBC), sodium dodecyl sulfate (SDS), normal human serum, IgG from human serum, sodium phosphate dibasic, N,N,N′,N′-tetramethylethylenediamine (TEMED), formic acid, sodium cyanoborohydride, acetic acid, and dimethyl sulfoxide were all purchased from Sigma-Aldrich. 2-Aminobenzamide (2-AB) was obtained from Alfa Aesar. Sodium chloride, sodium azide, potassium chloride, sodium phosphate monobasic, Tris-HCl, and glacial acetic acid (99.7%) were purchased from Fisher Scientific. Glycine was obtained from Calbiochem, and PNGase F from New England BioLabs. 6-Aminoquinolyl-N-hydroxysuccinimidyl carbamate was purchased from Biosynth Carbosynth. Whatman 3MM CHR chromatography paper was obtained from Cytiva. Ammonium peroxodisulfate (APS) was purchased from AnalaR (BDH 100323W). Protogel (30% acrylamide, 0.8% bis-acrylamide; 37.5:1) was purchased from National Diagnostics. Glycan preparation and release were carried out on a MicroLab Starlet liquid-handling system controlled using Venus Two software from Hamilton Bonaduz AG. Protein concentrations were measured using a UV-Vis Lambda 365 spectrophotometer from PerkinElmer. Adhesive oil was purchased from VWR, the PageRuler Prestained Protein Ladder from Thermo Scientific, and Mini-Protean TGX gels along with electrophoresis equipment (Mini-Protean Tetra Cell and PowerPac Basic) from Bio-Rad.

### 1.2. N-Glycan release and 2-AB labelling of serum using in-gel block method

*N*-glycan release and labelling from serum samples was performed using an in-gel block method (1). Serum samples (5 µL) were reduced by combining with 2 µL water, 2 µL sample buffer (100 µL 10 % SDS, 62.5 µL stacking buffer (0.5 M Tris pH 6.6) and 337.5 µL water), and 1 µL 0.5 M DTT, followed by gentle mixing and incubation at 65°C for 15 minutes. Alkylation was performed by adding 1 µL 100 mM IAA, mixing gently, and incubating for 30 minutes at room temperature in the dark. Polyacrylamide gel blocks were prepared by sequentially combining 22.5 µL Protogel, 11.25 µL gel buffer (1.5 M Tris pH 8.8), 1 µL 10% SDS, and 1 µL 10% APS with gentle mixing, followed by addition of 1 µL TEMED. After polymerization for 15 minutes, gels were sectioned into 1 mm³ pieces and transferred to an AcroPrep Nucleic Acid binding plate. The gel pieces were washed alternately with 1 mL acetonitrile and 1 mL 20 mM NaHCO₃ (10 minutes each with agitation) in the sequence: acetonitrile, NaHCO₃, acetonitrile, NaHCO₃, and acetonitrile. N-glycans were released by adding 50 μL PNGase F solution (1:400 in 20 mM NaHCO₃), followed by an additional 50 μL after 15 minutes, and 50 μL NaHCO₃ buffer, then incubated at 37°C overnight. Released glycans were eluted with three consecutive washes of 200 μL water, followed by acetonitrile, water, and acetonitrile (30 minutes each), then dried overnight in a vacuum centrifuge. Prior to labelling, samples were treated with 20 μL 1% formic acid for 40 minutes at room temperature and dried. The 2-AB labelling mixture was prepared by dissolving 484 mg NaBH₃CN in 5.376 mL DMSO and 369 mg 2-AB in 2.304 mL AcOH, then combining these solutions. Labelling was performed by adding 5 μL of the mixture to each sample, agitating for 5 minutes, incubating at 65°C for 30 minutes, agitating again, and incubating for an additional 1.5 hours at 65°C. Labelled samples were stored at -20°C for at least 5 minutes before clean-up. Excess 2-AB was removed using Whatmann 3MM paper and washed with 1.5 mL MeCN x7 and vortexed for 15 minutes. Lastly, 1.8 mL of water was used to wash the *N*-glycans that were dried and ready for UPLC analysis.

### 1.3. Automated IgG affinity purification from human serum and N-glycan release

The IgG anti-glycoprotein matrix was packed in PhyTips by PhyNexus Inc. In our case, Protein G PhyTips were used, with 20 µL of Protein G affinity resin packed into 300 µL Hamilton tips. The protocol was followed as previously described (2). To identify the optimal purification settings, we evaluated several different combinations of capture, binding, and elution cycles on the Hamilton Liquid Handling Station (MicroLab Star LET). Each setting consisted of a defined number of capture cycles, binding cycles, and elution cycles. Proteins were resolved by SDS-PAGE using Bio-Rad TGX precast gels (Supporting Figure S1), the condition consisting of 6 capture cycles, 6 binding cycles, and 3 elution cycles (6,6,3) produced the cleanest IgG bands. Although small traces of other serum glycoproteins can be carried through during affinity purification, as reported previously, their contribution in our case appears minimal based on the very faint non-IgG bands observed in the gel (2). Denaturation, alkylation, and enzymatic release of IgG *N*-glycans was performed according to the literature protocol (2) except for the following: *N*-glycans were released manually using 10KDa Nanosep cartridges, 0.5 M IAA was used for the alkylation, PNGase F treatment (0.416 µL, 25 mM SBC,  final concentration 208 U/mL) was performed at 37 °C for 1 hr followed by fluorescent labelling. A 40 µL volume of collected *N*-glycans was reacted with 100 µL aminoquinolyl-*N*-hydroxysuccinimidyl carbamate (AQC) (3 mg/mL) in MeCN. Excess label was cleaned using a HyperSep-96 Diol plate 100 mg Well Plate and 95 % MeCN, and the *N*-glycans were collected in 20 % MeCN. The final sample was dried down.

### 1.4. ^1^D TGX gel electrophoresis

15 mL of sample buffer (1 M Tris-HCL, 1 M DTT, 0.6 g SDS, 30 mg of Coomassie blue, 2.4 mL glycerol) was added to 10 mg of affinity purified glycoproteins and reduced at 95 °C for 6 min. Using a TGX Gel, 10 µL of the denatured samples and 5 µL protein ladder was loaded and separated using Mini Protean Tetra Cell (BioRad) for 30 min at 80V followed by a further 90 min at 120 V in 1x running buffer (25 mM Tris-Base, 192 mM glycine and 0.1 % (w/v) SDS, at pH 8). Once separation of proteins was visualised by staining with Coomassie Blue (0.1 % Coomassie Blue, 40 % ethanol and 10 % acetic acid) followed by destaining (10 % ethanol and 7.5 % acetic acid) (Supporting Figure S1).

### 1.5. Ultra Performance Liquid Chromatography (UPLC) for N-glycan visualisation and quantification

UPLC was performed as per literature report (3) and GU values were compared to those reported in (2). In short, the total serum 2AB and IgG AQC-derivatized N-glycans were visualised and quantified using UPLC and fluorescence detection on a Waters ACQUITY UPLC H-Class instrument consisting of a binary solvent manager, sample manager, and fluorescence detector, controlled by Empower 3 software (Waters, Milford, MA). The HILIC separation was performed using a Waters (BEH) Glycan column (130 Å, 1.7 µm particle, 2.1 mm x 150 mm) with 50 mM ammonium formate (pH 4.4) as solvent A and Acetonitrile (MeCN) as solvent B. The ACQUITY UPLC H-Class system was fitted with a 0.2 µm filter. Separation of 2-AB labelled total serum N-glycans and of AQC derivatized IgG N-glycans was performed using a linear gradient of 70-30% MeCN (0.56 mL/min, 30 min and 20 min respectively). An injection volume of 19 µL was prepared in 70 % v/v MeCN for each injection.

The FLD excitation/emission wavelength were λem= 420 nm and λex = 330 nm for 2AB and λem = 245 nm and λex = 395 nm for AQC. Samples were maintained at 5 °C before injection, and the column was set at 40 °C for separation for both methods. The system was calibrated using an external standard of 2-AB labelled dextran. A fifth-order polynomial distribution curve was fitted to the dextran ladder to assign glucose units (GU) values from retention times (using Empower software from Waters). The chromatograms obtained for total serum N-glycans were integrated for 46 glycan peaks (GPs) as per (4) and reported as percentages of total glycans (Supporting Table S1). For IgG N-glycan chromatograms were integrated for 23 GPs as per (2) and reported as a percentage of total glycans (Supporting Table S2 (2)). The characteristics of total serum and IgG N-glycans are calculated (Supporting Table S3).

### 1.6. Statistical analysis

All data processing and statistical analyses were performed using R version 4.5.1 (2025-06-13; “Great Square Root”) on a 64-bit Windows platform. The following R packages were used: readxl, dplyr, tidyr, stringr, readr, purrr, tibble, writexl, ggplot2, and knitr. Normality of each glycan variable was tested separately for the *Healthy* and *disease* groups using the Shapiro–Wilk test, data was log-transformed and normalised. P-values were obtained using ANOVA with Type I sums of squares (anova(fit) in R), and q-values were calculated using Benjamini–Hochberg correction across all glycan variables (Supporting Table S4). Significance stars displayed in boxplots represent unadjusted p-values from the corresponding regression models. For MELD-Na analyses, serum and IgG models were adjusted for age and sex. Boxplots and linear regression plots were generated using ggplot2 to visualise the effects of age, sex, and disease across the cohort. We examined associations between glycan features and MELD-Na using log10-transformed abundances. Spearman correlation coefficients (ρ) between each glycan and MELD-Na category (band vs. rest) were computed and visualised as a heatmap. Discriminatory ability of individual glycans was assessed using one-vs-rest ROC analyses to obtain AUC values, and effect sizes were estimated using Cohen’s d. A composite fingerprint score (z-normalised Spearman, AUC, and Cohen’s d) was then used to identify the top discriminative glycans within each MELD-Na range.

## Supporting Figures

10

9

8

7

6

5

4

3

2

1

KDa

KDa

**Figure S1**. Zoom in of TGX-gel electrophoresis which was used to compare different capture, wash, and elution cycle configurations for optimisation of IgG isolation from normal human serum (NHS). All conditions use approximately 8 µg of protein. Lane 1: Ladder. Lane 2 is an IgG standard. Lane 3: **3,3,3** method. Lane 4: **3,3,3 x2** (samples isolated two times). Lane 5: **6,6,3** method. Lane 6: **6,6,3 x2** (sample isolated two times). Lane 7: **9,3,3** method. Lane 8: **9,6,3** method. Lane 9: **12,6,3** method. Lane 10: Ladder. The 3,3,3 indicates 3 protein captures, 3 washes and 3 elution aspirations.

15

**Figure S2**. Linear regression showing the effects of age on serum N-glycome (GP1-46) and glycan-derived traits in alcohol-related liver disease (ALD, n = 17, age 57 ± 7), primary sclerosing cholangitis (PSC, n = 7, age 56 ± 9), and hepatocellular carcinoma (HCC, n = 4, age 57 ± 9).

**Figure S3.** Linear regression showing the effects of age on IgG N-glycome (GP1–23) and glycan-derived traits in alcohol-related liver disease (ALD, n = 17, age 57 ± 7), primary sclerosing cholangitis (PSC, n = 7, age 56 ± 9), and hepatocellular carcinoma (HCC, n = 4, age 57 ± 9).

**Figure S4**. Boxplots comparing serum N-glycome (GP1-46) and glycan-derived traits between males and females in ALD (males = 11, females = 6), PSC (males = 5, females = 2), and ALD-derived HCC (4 males).

**Figure S5**. Boxplots comparing IgG N-glycome (GP1-23) and glycan-derived traits between males and females in ALD (males = 11, females = 6), PSC (males = 5, females = 2), and ALD-derived HCC (4 males).

**Figure S6.** Boxplots showing the comparison of serum N-glycan peaks (GP1-46) and glycan-derived traits between healthy (n = 10), alcohol-related liver disease (ALD, n = 17), ALD derived hepatocellular carcinoma (HCC, n = 4) and primary sclerosing cholangitis (PSC, n = 7), and hepatocellular carcinoma (HCC, n = 4), adjusted for age and sex. The stars above the boxes represent per-subgroup vs healthy (*** <0.01, ** <0.025 and * <0.05 significant p values). The p-values and q-values are presented in Supporting Table S4.

**Figure S7.** Boxplots showing the comparison of IgG N-glycan peaks (GP1-23) and glycan-derived traits between healthy (n = 9), ALD (n = 17), ALD-derived HCC (n = 4) and PSC (n = 7), adjusted for age and sex. The stars above the boxes represent per-subgroup vs healthy (*** <0.01, ** <0.025 and * <0.05 significant p values). The p-values and q-values are presented in Supporting Table S5.

**Figure S8.** Linear regression of individual IgG glycan peaks (GP1-GP23) and derived glycan traits in ALD in relation to MELD-Na score. Regression lines represent age- and sex-adjusted linear models. Glycans highlighted in orange correspond to features ranked among the strongest MELD-Na associations based on the magnitude of their regression slopes (positive or negative). Full regression statistics, including effect sizes and Benjamini-Hochberg FDR-adjusted q-values, are reported in Supporting Table S6.

**Figure S9.** Age-associated regression analysis of serum N-glycan derived traits in an age-matched subgroup of healthy controls and liver disease patients. The regression line shows the relationship between age and 18 derived serum N-glycan traits in an age-overlapping subgroup comprising healthy controls aged 45–51 years (n=4), ALD patients aged 45–55 years (n=7), and PSC patients aged 45–55 years (n=4). This analysis was performed to confirm that age-related glycomic trends observed in the full cohort remain directionally consistent when restricted to the age-overlapping subset.

**References**

1.	Royle L, Campbell MP, Radcliffe CM, White DM, Harvey DJ, Abrahams JL, et al. HPLC-based analysis of serum N-glycans on a 96-well plate platform with dedicated database software. Anal Biochem. 2008;376(1):1–12.

2.	O'Flaherty R, Muniyappa M, Walsh I, Stöckmann H, Hilliard M, Hutson R, et al. A Robust and Versatile Automated Glycoanalytical Technology for Serum Antibodies and Acute Phase Proteins: Ovarian Cancer Case Study. Mol Cell Proteomics. 2019;18(11):2191–206.

3.	Stöckmann H, Duke RM, Millán Martín S, Rudd PM. Ultrahigh throughput, ultrafiltration-based n-glycomics platform for ultraperformance liquid chromatography (ULTRA(3)). Anal Chem. 2015;87(16):8316–22.

4.	Saldova R, Asadi Shehni A, Haakensen VD, Steinfeld I, Hilliard M, Kifer I, et al. Association of N-Glycosylation with Breast Carcinoma and Systemic Features Using High-Resolution Quantitative UPLC. Journal of Proteome Research. 2014;13(5):2314–27.
